# Chronic fluoxetine treatment reduces parvalbumin expression and perineuronal nets in gamma-aminobutyric acidergic interneurons of the frontal cortex in adult mice

**DOI:** 10.1186/1756-6606-6-43

**Published:** 2013-11-05

**Authors:** Koji Ohira, Rika Takeuchi, Tsuyoshi Iwanaga, Tsuyoshi Miyakawa

**Affiliations:** 1 Division of Systems Medical Science, Institute for Comprehensive Medical Science, Fujita Health University, Toyoake 470-1192, Japan; 2 Core Research for Evolutional Science and Technology, Japan Science and Technology Agency, Kawaguchi 332-0012, Japan; 3 Section of Behavior Patterns, Center for Genetic Analysis of Behavior, National Institute for Physiological Sciences, Okazaki 444-8585, Japan

**Keywords:** Antidepressant, Calcium-binding protein, Depression, Emotion, Extracellular matrix, GABA, Interneuron, Limbic system, Parvalbumin, Prelimbic cortex

## Abstract

**Background:**

The selective serotonin reuptake inhibitor fluoxetine (FLX) is widely used to treat depression and anxiety disorders, but cellular mechanisms underlying the antidepressant effect of FLX remain largely unknown. The generally accepted effect of chronic FLX treatment is increased adult neurogenesis in the hippocampal dentate gyrus. It was recently demonstrated that FLX treatments can reverse the established neuronal maturation of granule cells in the hippocampal dentate gyrus and of gamma-aminobutyric acidergic (GABAergic) interneurons in the basolateral amygdala. However, it is not clear whether this dematuration effect of FLX occurs in other brain regions. Thus, in this study, we used immunohistological analysis to assess the effect of FLX treatment on GABAergic interneurons in the medial frontal cortex (mFC) and reticular thalamic nucleus (RTN).

**Results:**

Immunofluorescence analysis for perineuronal nets (PNNs), which is a marker of neuronal maturation, and for parvalbumin, calretinin, and somatostatin, which are markers for specific GABAergic interneuron type, showed lower number of parvalbumin-positive (+) cells and PNN+/parvalbumin+ cells in the mFC of FLX-treated mice compared to vehicle-treated mice. However, FLX treatment had no effect on the number of cells expressing calretinin and somatostatin in the mFC. In the RTN, the number of PNN+ cells and parvalbumin+ cells was unaltered by FLX treatments. Furthermore, the number of total GABA+ cells and apoptotic cells in the mFC was similar between vehicle- and FLX-treated mice, suggesting that FLX treatment did not induce cell death in this region. Rather, our findings suggest that the decreased number of parvalbumin+ cells in the mFC was due to a decreased expression of parvalbumin proteins in the interneurons.

**Conclusions:**

This study indicates that FLX decreases the levels of parvalbumin, a mature marker of fast-spiking interneurons, and PNNs in parvalbumin+ interneurons in the mFC, suggesting that FLX treatment induces a dematuration of this type of neurons. Induction of a juvenile-like state in fast-spiking inhibitory interneurons in these regions might be involved in the therapeutic mechanism of this antidepressant drug and/or some of its adverse effects.

## Background

Fluoxetine (FLX), a selective serotonin reuptake inhibitor (SSRI), is widely used to treat depressive disorder; however, the cellular mechanisms underlying the antidepressant effect of FLX remain unclear. Findings from animal studies suggest that adult neurogenesis in the brain is critically involved in this process [1]. It has been reported that chronic FLX treatment for 2–4 weeks results in increased neurogenesis and cell proliferation in the adult dentate gyrus (DG) [2-4], a response that has been linked to the behavioral effects of FLX [3]. Furthermore, we recently demonstrated that chronic FLX treatment leads to the generation of cortical gamma-aminobutyric acidergic (GABAergic) interneurons from neural progenitor cells in adult mice [5]. Conversely, we have shown that chronic FLX treatment for more than 6 weeks decreases neurogenesis in the subventricular zone of adult mice [6].

Besides its effect on adult neurogenesis, chronic FLX treatments cause “dematuration,” a reversal of the established state of maturation of adult dentate granule cells [6-9], raising the possibility that a distinct form of synaptic plasticity underlies the antidepressant effect of FLX. Dentate granule cells in FLX-treated adult mice exhibit similarity with immature granule cells in non-treated mice in terms of expressions of maturation cell markers (e.g., a decrease in calbindin expression and an increase in calretinin expression) and electrophysiological characteristics (e.g., reductions of basal synaptic transmission and frequency facilitation of the synapses between DG and CA3, and reinstatement of high membrane excitability) [7]. A juvenile-like state of granule cells has also been observed in the adult brains of some genetically-engineered mice strains [10], such as alpha calcium/calmodulin-dependent protein kinase II (αCaMKII) heterozygous knockout (HKO) mice [11-17], schnurri-2 (Shn-2) KO mice [18], and mutated synaptosomal-associated protein 25 knock-in (SNAP-25 KI) mice [19]. Interestingly, FLX-induced dematuration of neurons has also been observed in GABAergic interneurons of the basolateral amygdala [20]; FLX converts interneurons, in particular parvalbumin-positive (+) cells, a subclass of interneurons, to a more immature state. However, it remains unclear whether FLX has any effect on cellular dematuration of interneurons in other brain regions.

In this study, we used immunohistological analysis to investigate the effect of FLX treatment on GABAergic interneurons of the medial frontal cortex (mFC) and reticular thalamic nucleus (RTN). Specifically, we assessed whether FLX treatment demonstrated a dematuration effect on GABAergic interneurons by examining the expression of perineuronal net (PNN), a marker of neuronal maturation, as well as the expression of parvalbumin, calretinin, and somatostatin, which are markers for specific GABAergic interneurons.

## Results

### Chronic FLX treatment decreased the number of parvalbumin+ cells in the frontal cortex

PNNs are reticular structures composed of extracellular matrix molecules, such as chondroitin sulfate proteoglycans, hyaluronan, and tenascin-R, and are expressed in the central nervous system [21]. The temporal pattern of PNN formation reportedly corresponds to the ending of the critical periods in which synaptogenesis, synaptic refinement, and myelination occur [21], thus suggesting that their formation coincides with neuronal maturation. For this reason, PNNs are considered a marker of neuronal maturation [20,22,23].

**Figure 1:**
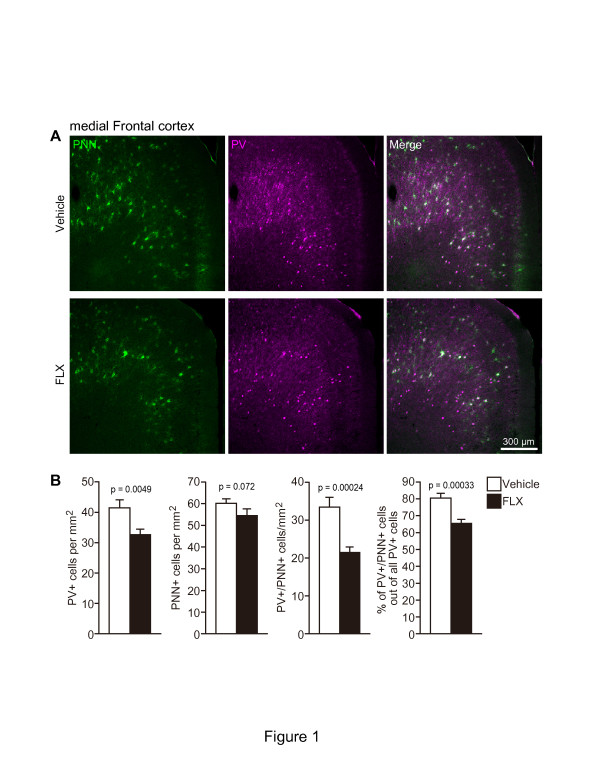
**Decrease in the number of parvalbumin+ cells in the mFC. (A)** Representative coronal images of parvalbumin+ (magenta)/PNN+ (green) cells in the mFC of mice treated with vehicle (upper row) or FLX (lower row). Mice received FLX for 3 weeks at 15 mg/kg/day. **(B)** Quantification of the number of parvalbumin+, PNN+, parvalbumin+/PNN+ cells, and the proportion of parvalbumin+/PNN+ cells in the total number of parvalbumin+ cells (n = 4 mice each; 11-week-old). FLX, fluoxetine; PNN, perineuronal net; PV, parvalbumin.

Using PNN and parvalbumin stained sections, we first examined whether chronic FLX treatment altered the staining pattern of parvalbumin+ cells in the mFC of adult mice. FLX solution was intraperitoneally injected into mice at 15 mg · kg^-1^ · day^-1^ for 3 weeks. We chose the mFC (Additional file 1: Figure S1), because it extensively overlaps with regions referred to as the anterior cingulate cortex [24] and because it plays an important role in rodent emotional memory and behavior associated with limbic regions, such as the amygdala and hippocampus [25-27]. The number of parvalbumin+ cells in the mFC was significantly decreased in FLX-treated mice compared to vehicle-treated mice (Figure 1; p = 0.0049). There was no significant difference in the number of total PNN+ cells between FLX- and vehicle-treated mice, although the number of PNN+ cells tended to decrease by chronic FLX treatment (p = 0.072). Chronic FLX treatment significantly decreased the number of parvalbumin+/PNN+ cells (p = 0.00024). Using these data, we calculated the percentage of parvalbumin+/PNN+ cells from the total number of parvalbumin+ cells and found that FLX treatment decreased the percentage of parvalbumin+/PNN+ cells to approximately 80% of the vehicle-treated value (p = 0.00033).

**Figure 2:**
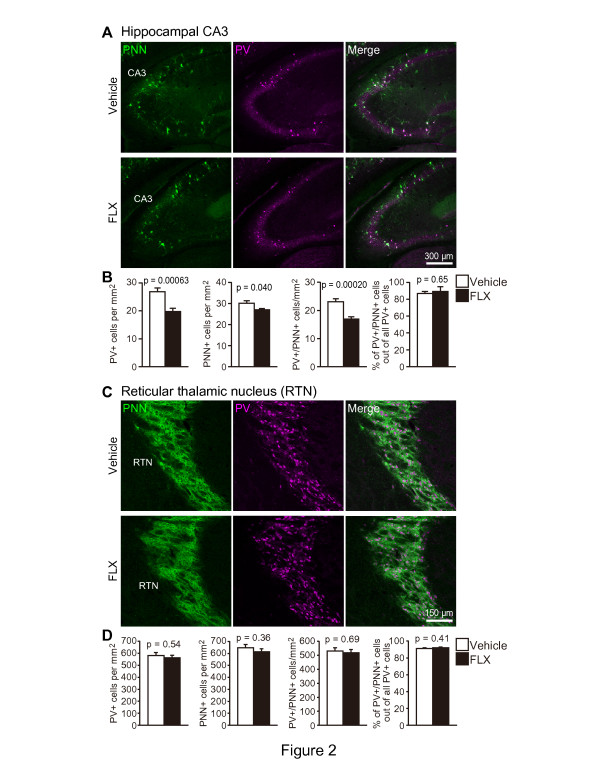
**Expression of parvalbumin and PNN in the hippocampal CA3 and RTN. (A, C)** Double-staining for parvalbumin (magenta) and PNN (green) in coronal sections of the hippocampal CA3 region **(A)** and RTN **(C)**. Mice received FLX for 3 weeks at 15 mg/kg/day. **(B, D)** The number of parvalbumin+, PNN+, parvalbumin+/PNN+ cells, and the proportion of parvalbumin+/PNN+ cells in the total number of parvalbumin+ cells were quantified in the hippocampal CA3 region **(B)** and RTN **(D)** (n = 4 mice each; 11-week-old). Note that a decreased number of parvalbumin+ cells were found in the hippocampal CA3, but not in the RTN. CA, cornu ammonis; FLX, fluoxetine; PNN, perineuronal net; PV, parvalbumin; RTN, reticular thalamic nucleus.

We also investigated whether FLX treatment affected the number of parvalbumin+ cells in the RTN. In the RTN, in which almost all neurons are GABAergic, FLX treatment had no effect on the numbers of parvalbumin+ (p = 0.54) and PNN+ cells (p = 0.36) (Figure 2C and D). However, we found a significantly lower number of parvalbumin+ cells in the hippocampal CA3 region of FLX-treated mice compared to vehicle-treated mice (Figure 2A and B; p = 0.00063). The number of PNN+ cells was also significantly decreased in the hippocampal CA3 region (Figure 2A and B; p = 0.040) but not in the RTN. FLX treatment also reduced the number of parvalbumin+ cells in the basolateral amygdala (Additional file 1: Figure S2; p = 0.0058). Our results from the hippocampal CA3 region and basolateral amygdala corroborate previous findings [20].

### Chronic FLX treatment did not alter the numbers of calretinin+ and somatostatin+ cells

Three main subgroups of GABAergic interneurons are found in the FC of adult rodents: parvalbumin+ cells, calretinin+ cells, and somatostatin+ cells [28]. Thus, we next examined whether chronic FLX treatment decreased the expression of calretinin and somatostatin in the mFC, RTN, hippocampus, and basolateral amygdala. Chronic FLX treatment had no effect on the number of calretinin+ and somatostatin+ cells in these brain regions (Additional file 1: Figure S3 and Table S1). In addition, the calretinin+ and somatostatin+ cells in the mFC, RTN, hippocampus, and basolateral amygdala were not surrounded by PNNs (Additional file 1: Figure S3 and Table S1), which is consistent with previous findings [29,30]. Although calbindin was abundantly expressed in the RTN, FLX treatment did not alter its expression (Additional file 1: Figure S3 and Table S1). This result suggests that FLX treatment mainly affected parvalbumin+ interneurons, and not calretinin and somatostatin, in the mFC, hippocampus, and basolateral amygdala. It also highlights that compared to calretinin+ and somatostatin+ cells, parvalbumin+ cells are more likely to be surrounded by PNNs.

### Chronic FLX treatment did not alter the numbers of GABA+ cells and apoptotic cells

**Figure 3:**
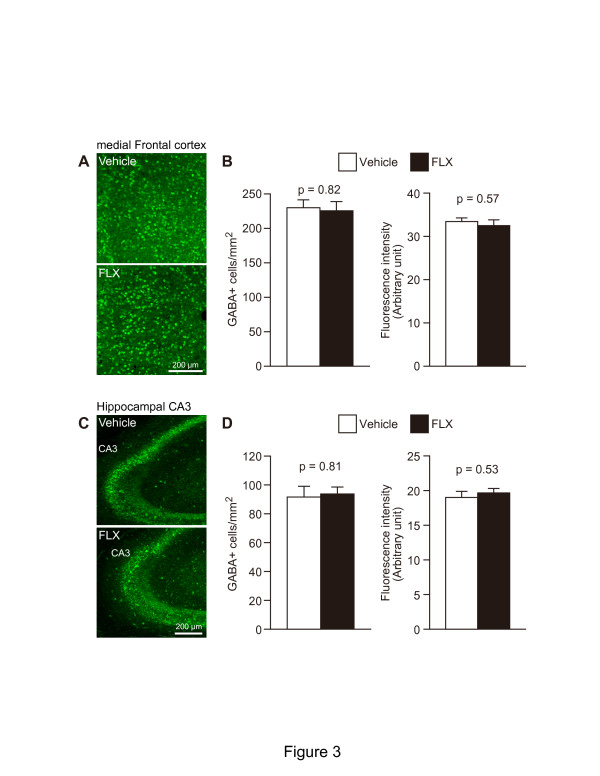
**FLX treatment did not change the number of GABA+ cells in the mFC and hippocampus. (A, C)** Representative coronal images of GABA+ cells in the mFC **(A)** and hippocampus **(C)** of vehicle-treated (upper) and FLX-treated mice (lower). Mice received FLX for 3 weeks at 15 mg/kg/day. **(B, D)** The number of GABA+ cells (left graphs) and the fluorescence intensity of GABA (right graphs) were quantified in the mFC **(B)** and hippocampal CA3 region **(D)** of vehicle-treated and FLX-treated mice (n = 4 mice each; 11-week-old). CA, cornu ammonis; FLX, fluoxetine; mFC, medial frontal cortex.

Although we had observed a significant decrease in the number of parvalbumin+ cells in the mFC and hippocampus of FLX-treated mice, it remained unclear whether this decrease was reflective of parvalbumin+ interneuron cell death or a decrease in the expression of parvalbumin proteins in each interneuron. Thus, we performed immunofluorescence staining for GABA to examine the effect of FLX treatment on the total numbers of GABAergic interneurons in these regions. We found no difference in the number of GABA+ cells in the mFC (Figure 3A and B; p = 0.82) and hippocampus (Figure 3C and D; p = 0.81) of FLX-treated mice compared to vehicle-treated mice. Furthermore, the fluorescence intensity of GABA in the mFC and hippocampus of FLX-treated mice was similar to that in these regions of vehicle-treated mice (Figure 3B and D; mFC, p = 0.57; Hippocampal CA3, p = 0.53). We subsequently examined whether the number of apoptotic cells changed after FLX treatments. Terminal deoxynucleotidyl transferase-mediated dUTP nick-end labeling (TUNEL) analysis revealed that FLX treatments did not induce apoptotic cell death in the mFC and hippocampus (Additional file 1: Figures S4). In addition, we labeled the interneurons with 5-bromodeoxyuridine (BrdU), a marker of DNA synthesis, by intraperitoneal injection of BrdU into timed-pregnant mice every 24 h from day 14 to day 20 of gestation. Thus, the interneurons generated during the embryonic period contained BrdU. FLX treatment was commenced 8 weeks after birth and continued for 3 weeks. FLX treatment did not alter the number of BrdU+ cells in the mFC or hippocampus; however, it significantly reduced the number of parvalbumin+/BrdU+ cells (Additional file 1: Figures S5 and S6). This result suggests that, in the cells generated during embryogenesis, parvalbumin protein levels in the mFC and hippocampus were reduced after FLX treatment. Taken together, these findings suggest that the decreased number of parvalbumin+ cells in the mFC and hippocampus may reflect a decreased expression of parvalbumin proteins in each interneuron.

## Discussion

In this study, we examined the effect of chronic FLX treatment on PNN expression as well as the expression of parvalbumin+, calretinin+, and somatostatin+ cells in the mFC, hippocampus, basolateral amygdala, and RTN. Immunofluorescence analysis revealed that FLX treatment decreases the number of parvalbumin+ cells, but not that of calretinin+ or somatostatin+ cells, in the mFC, hippocampus, and basolateral amygdala. Our findings suggest that the decreased number of parvalbumin+ cells reflects a decrease in parvalbumin protein expression in each interneuron, and not apoptotic cell death of parvalbumin+ cells or a decrease in the total number of GABA+ cells. Furthermore, the percentage of parvalbumin+/PNN+ cells was also decreased in the mFC. These findings suggest that FLX treatment may have a dematuration effect on fast-spiking inhibitory interneurons in the mFC and hippocampus, which are immunoreactive for parvalbumin during the mature status [31]. This pseudo-immature state of parvalbumin+ cells may account for the antidepressant effect of FLX, in addition to, or as an alternative to, dematuration in the DG and amygdaloid neurons and increased neurogenesis in the DG and cortex.

### Decreased percentage of PNN+ cells out of parvalbumin+ cells in the mFC

In the present study, we found a decrease in the number of PNN+ cells in the hippocampal CA3 region and a decrease in the proportion of PNN+/parvalbumin+ cells in the total number of parvalbumin+ cells in the mFC, basolateral amygdala, and hippocampal CA3 region. It has previously been demonstrated that chronic FLX treatment decreases the percentage of PNN+/parvalbumin+ cells (from the total number of parvalbumin+ cells) in the basolateral amygdala and hippocampal CA1 [20]; however, to our knowledge, such a finding has not been observed in the FC until now. In contrast to our study, a previous study reported no disruption to PNNs in the FC [20]. This difference may be attributable to the differences in FLX administration, such as the dose of FLX (10 mg · kg^-1^ ∙ day^-1^ in the previous study vs. 15 mg ∙ kg^-1^ ∙ day^-1^ in this study) and administration method (drinking water in the previous study vs. intraperitoneal injection in this study). This discrepancy in findings for PNN expression in the mFC should be examined in future studies to determine whether it is a dose-dependent effect of FLX.

The formation of PNNs coincides with neuronal maturation in the central nervous system [20,22,23]. Thus, similar to other studies, we used PNN as a marker of neuronal maturation. We found a significant decrease in PNN+ cells in the hippocampal CA3 region following FLX treatment; however, FLX treatment had less effect on the total number of PNN+ cells in the mFC. This is probably because the reduction of PNN happens specifically in the parvalbumin+ cells in the mFC. Also, it may be due to the long life of PNN components. It has previously been demonstrated that the immunoreactivity of PNN components, tenascin and chondroitin sulphate proteoglycans, persists *in vivo* for at least up to 4 weeks and 14 months, respectively [32]. In this study, we used *Wisteria floribunda* lectin (WFA) to detect PNNs. WFA binds carbohydrate structures terminating in *N*-acetylgalactosamine linked to galactose, which are contained in chondroitin sulphate proteoglycans of PNNs [32]. Thus, this suggests that dematurated interneurons are still surrounded by PNNs or chondroitin sulphate proteoglycans after the disappearance of parvalbumin proteins in each interneuron. Consequently, fast-spiking cells, in which parvalbumin proteins are diminished by FLX treatment, can be detected by WFA.

No significant differences were observed in the numbers of parvalbumin+ or PNN+ cells in the RTN between vehicle- and FLX-treated mice. Currently, it is not clear why these numbers did not change after FLX treatment. However, possibly, this could be attributable to the origins of the GABAergic interneurons present in the regions. Almost all the cortical, hippocampal, and amygdaloid GABAergic interneurons are derived from the ventricular zone of the medial and caudal ganglionic eminences [33,34], while thalamic GABAergic interneurons originate from the ventricular zone of the third ventricles [35]. These results suggest the possibility that FLX treatment might specifically reduce parvalbumin protein levels in interneurons derived from the ganglionic eminences. It will be interesting to examine this, as well as other possibilities, in future research.

### Decrease in the number of parvalbumin+ cells in the mFC and hippocampus

In this study, we demonstrated that chronic FLX treatment did not induce apoptotic cell death of parvalbumin+ interneurons in the mFC and hippocampus. We also showed that the number of GABA+ cells in the mFC and hippocampus was not altered by FLX treatment. These findings suggest that the decreased number of parvalbumin+ cells reflects a decrease in expression of parvalbumin proteins in each cell.

**Figure 4:**
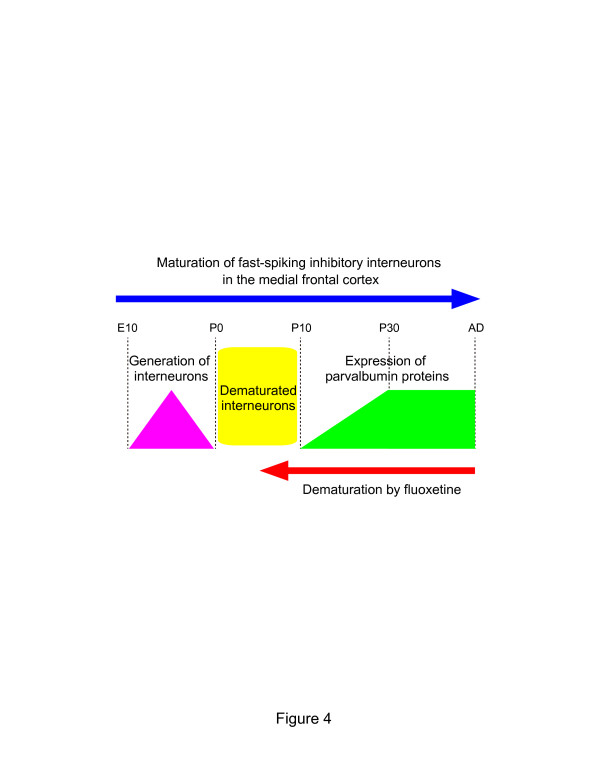
**Dematuration of cortical fast-spiking inhibitory interneurons by FLX treatments.** Based on parvalbumin expression, the state of the dematurated fast-spiking inhibitory interneurons in the mFC of FLX-treated mice were similar to that of fast-spiking inhibitory interneurons of non-treated mice between postnatal days 0 and 10 (indicated in yellow). E, embryonic day; P, postnatal day.

Three main subgroups of GABAergic interneurons are found in the FC of adult rodents: parvalbumin+, calretinin+, and somatostatin+ cells [28]. On the other hand, based on the firing patterns for depolarization, cortical GABAergic interneurons are divided into three subgroups: fast-spiking, late-spiking, and regular-spiking/burst-spiking non-pyramidal cells [36]. It is widely accepted that almost all fast-spiking interneurons express parvalbumin, whereas interneurons with the other types of spiking patterns have calretinin and somatostatin [36]. In rodents, almost all fast-spiking inhibitory GABAergic interneurons in both cortex [37] and hippocampus [38] are generated during the embryonic period, while the first parvalbumin proteins appear at postnatal day 10 in the mouse cortex [39] and at postnatal day 7 in the hippocampus [40]. Moreover, using transcriptional and electrophysiological analyses of a GFP knock-in mice, in which almost all fast spiking inhibitory interneurons express GFP, fast-spiking inhibitory interneurons have been reported to mature between P10 and P25 [31,41]. Thus, immature fast-spiking inhibitory interneurons in the cortex do not express parvalbumin mRNA. The present result suggests that FLX treatment may convert mature parvalbumin+ interneurons to a pseudo-immature state, i.e., FLX treatment may cause “dematuration” of parvalbumin+ interneurons (Figure 4).

### Implication of FLX-induced neuronal dematuration in the mFC, hippocampus, and amygdala

In adult mice, FLX treatment converts differentiated DG neurons to a more immature state [6-9]. Similar changes in DG neurons have been demonstrated in αCaMKII HKO [11], Shn-2 KO [18], and SNAP-25 KI mice [19]; this phenomenon has been termed the “immature DG” [10]. In this study, we demonstrate for the first time that FLX treatment might also induce dematuration of parvalbumin+ interneurons in the mFC and hippocampal CA3 region, while our finding in the amygdala is consistent with that of a previous study [20]. Consistently, the present study demonstrates that chronic FLX treatment increases the expression of polysialic acid-neural cell adhesion molecule (PSA-NCAM), which is a marker for immature neurons and a regulator of neural plasticity [42,43], in the mFC, hippocampus, and amygdala; this is in agreement with previous findings [44,45] (Additional file 1: Figure S7). This suggests that dematuration of parvalbumin+ interneurons is induced by FLX treatment in the mFC, hippocampus, and amygdala, where neural plasticity might be enhanced by FLX treatment. In line with this, FLX treatment has been reported to reinstate neural plasticity and promote the electrophysiological and behavioral recovery of functions in the visual cortex of adult amblyopic rats [46]. In contrast, accelerated maturation of parvalbumin+ cells via overexpression of the neurotrophin brain-derived neurotrophic factor leads to a reduced capacity for cortical neural plasticity [47,48]. Thus, dematuration of parvalbumin+ interneurons in the mFC, hippocampus, and amygdala might reinstate synaptic plasticity that is reduced with age and development, thereby potentially causing the antidepressant effect of FLX. Further studies are required to address the causal relationship between dematuration of parvalbumin+ cells and enhanced neural plasticity.

Recent findings have led to the hypothesis that problems in information processing within neural networks, rather than altered chemical balance, may account for the mechanism underlying depression [49,50]. Thus, antidepressant drugs may induce changes in neuronal morphology and connectivity, gradually improving neuronal information processing and recovering mood. Indeed, volume changes in the hippocampus, mPFC, or amygdala are found both in patients with depression and in animal models of depression [51,52]. Previous studies, as well as the present one, have identified some of the effects of FLX on the brain, which include increased adult neurogenesis in the DG [2] and cortex [5], decreased adult neurogenesis in the SVZ [6], dematuration of neurons in the DG [7], amygdala [20], and mFC. Most events occur in the FC and limbic system. Interestingly, it has become increasingly clear that network dysfunction in the PFC and limbic system, including the hippocampus and amygdala, is involved in the pathophysiology of depressive disorder [24,53,54]. Therefore, neuronal dematuration and adult neurogenesis in these regions may play important roles in the mechanism of action of antidepressant drugs like FLX. In addition, some of the adverse effects of FLX [55], such as aggression, violence, and psychosis, might be mediated by the dematuration of fast-spiking inhibitory interneurons in the mFC. Aggression and violence are associated with deficits in the prefrontal cortex of humans [56,57], where activation of GABAergic interneurons decreases [57]. Dematuration of fast-spiking inhibitory interneurons might decrease inhibitory transmission of the interneurons, which in turn could evoke aggression and violence. It should be noted that, in post-mortem brains of patients with schizophrenia, the number of parvalbumin+ interneurons [41,58] and PNN+ cells [59] is decreased in the prefrontal cortex. This dematuration of parvalbumin+ fast-spiking interneurons by FLX treatment may be related to the antidepressant-induced psychosis and agression observed in clinical settings [55,60]. Future studies will need to address the behavioral significance of the FLX-induced dematuration effect on fast-spiking inhibitory interneurons in the mFC.

## Conclusions

The present study demonstrates that chronic FLX treatment reduces parvalbumin proteins and PNNs in GABAergic interneurons in the mFC, suggesting that FLX induces juvenile-like state of fast-spiking inhibitory interneurons in mFC. This effect of FLX on parvalbumin+ cells in the mFC might account for the therapeutic mechanism of this antidepressant drug and/or some of its adverse effects.

## Methods

### Animals and antidepressant treatment

Adult male C57BL/6 J mice (Charles River Laboratories Japan, Yokohama, Japan), that were 2 months old at the start of our experiments, were used. All animal experiments were approved by the Institutional Animal Care and Use Committee of Fujita Health University, based on the Law for the Humane Treatment and Management of Animals (2005) and the Standards Relating to the Care and Management of Laboratory Animals and Relief of Pain (2006). Every effort was made to minimize the number of animals used.

Treatment with FLX (LKT Laboratories, St. Paul, MN) was performed as previously described [5]. Briefly, FLX solution was intraperitoneally injected into mice between 10:00–11:00 a.m. every day for 3 weeks. The appropriate FLX concentration (15 mg ∙ kg^-1^ ∙ day^-1^) was determined for each body weight before injection. Mice were fixed at 6 h after the last injection of FLX. Chronic FLX treatment at this concentration remarkably decreased the expression of calbindin in the DG (Additional file 1: Figure S8; p = 0.00046), as previously reported [6,7].

### BrdU labeling

BrdU injection was performed as previously described [14]. Briefly, the BrdU (Sigma-Aldrich, St. Louis, MO) stock solution was prepared in distilled water with 0.007 N NaOH at 20 mg/ml and stored at −20°C until use. In order to label GABAergic interneurons of the embryonic cerebral cortex with BrdU, timed-pregnant mice were intraperitoneally injected with BrdU solution (100 mg/kg body weight) dissolved in phosphate buffered saline (PBS) every 24 h from day 14 to day 20 of gestation. After birth, the mice were bred for 2 months before subsequently receiving FLX injections for 3 weeks at a concentration at 15 mg ∙ kg^-1^ ∙ day^-1^. The mice were deeply anesthetized and transcardially perfused with 4% paraformaldehyde in 0.1 M phosphate buffer (PB), pH 7.4. For staining BrdU staining, sections were pretreated with HCl as previously described [14].

### Immunohistological analysis

Fixation and immunofluorescence staining were performed as previously described [5]. Briefly, mice were deeply anesthetized with chloral hydrate (245 mg/kg, intraperitoneally) and transcardially perfused with 4% paraformaldehyde in 0.1 M PB. The brains were dissected, immersed overnight in the same fixative, and transferred to 30% sucrose in PBS for at least 3 days for cryoprotection. All brain samples were mounted in Tissue-Tek (Miles, Elkhart, IN), frozen, and cut coronally into 50-μm-thick coronal sections, using a microtome (CM1850, Leica Microsystems, Wetzlar, Germany). Sections were stored in PBS containing sodium azide (0.05%, w/v) at 4°C until use. After washing in PBS for 1 h, they were preincubated with PBS-DB (4% normal donkey serum [Vector Laboratories, Burlingame, CA] and 1% BSA in PBS) for 2 h at room temperature. The sections were incubated at 4°C for 48 h or at room temperature overnight with the indicated primary antibodies. After washing in PBS for 1 h, the sections were incubated at room temperature for 1 h with secondary antibodies. For PNN staining, the sections were incubated with biotinylated WFA (1:200; Sigma-Aldrich) at 4°C for 48 h or at room temperature overnight. After washing in PBS for 1 h, the sections were incubated with Alexa Fluor 488 conjugated to streptavidin (10 μg/ml; Life technologies, Carlsbad, CA) for 1 h at room temperature. After washing in PBS containing Hoechst 33258 for nuclear counterstaining for 1 h, the sections were mounted on glass slides coated with 3-aminopropyltriethoxysilane and embedded with Permafluor (Thermo Scientific, Pittsburgh, PA). Confocal laser-scanning microscopy (LSM 700; Carl Zeiss, Oberkochen, Germany) was used to obtain images of the stained sections.

### Antibodies

The following primary antibodies were used: mouse monoclonal antibodies for calbindin (1:2000, Sigma-Aldrich), parvalbumin (1:2000, Sigma-Aldrich), PSA-NCAM (clone 2-2B mouse IgM; 1:200, Millipore, Billerica, MA); rat monoclonal antibody for BrdU (1:100, Abcam, Cambridge, MA); and rabbit polyclonal antibodies for calretinin (1:500, Life technologies), GABA (1:1000, Sigma-Aldrich), and somatostatin (1:1000, Bachem, Bubendorf, Switzerland). The following secondary antibodies were used: goat anti-mouse IgG Alexa Fluor 488 (1:200, Life Technologies), goat anti-mouse IgG Alexa Fluor 594 (1:200, Life Technologies), goat anti-mouse IgM Cy3 (1:200, Millipore), goat anti-rabbit IgG Alexa Fluor 594 (1:200, Life Technologies), and goat anti-rat IgG Alexa Fluor 594 (1:200, Life Technologies).

### TUNEL staining

TUNEL staining was performed according to the manufacturer’s instructions (*in situ* cell death detection kit, Roche, Mannheim, Germany).

### Ischemia treatment

Global ischemia was induced as previously described [61]. Briefly, after anesthesia, both common carotid arteries were transiently occluded with clamps for 10 min. Control animals were treated identically, except for the occlusion of common carotid arteries. The mice were allowed to survive for 2 days after ischemia and were then killed by perfusion.

### Quantification of labeled cells

The mFC region was determined according to the mouse brain atlas [62]. Quantification analysis was performed as previously reported [5]. Briefly, analysis was performed using a confocal microscope equipped with a 40 × objective lens (Plan-NEOFLUAR, NA = 0.75, Carl Zeiss) and a pinhole setting that corresponded to a focal plane thickness of less than 1 μm. To exclude false-positives due to the overlay of signals from different cells, randomly selected areas were analyzed by moving through the entire z-axis of each area. Cells were counted under the live mode of confocal scanning. For quantifying the fluorescence intensity of immunostained images, we used the ImageJ software. The region of interest of the acquired images was traced, and optical densities were obtained from at least three sections per mouse. Background intensity was subtracted using nonstained portions of each section. Slides were coded and quantified by a blinded independent observer. Data were analyzed by one-way ANOVA. The error bars in the figures represent SEM.

## Abbreviations

ACC: Anterior cingulate cortex; BrdU: 5-bromodeoxyuridine; DG: Dentate gyrus; FC: Frontal cortex; FLX: Fluoxetine; PFC: Prefrontal cortex; PNN: Perineuronal net; RTN: Reticular thalamic nucleus; SSRI: Selective serotonin reuptake inhibitor.

## Competing interests

Tsuyoshi Miyakawa is an advisor/consultant for Astellas Pharma Inc. The other authors declare no conflicts of interest.

## Authors’ contributions

KO and TM conceived the study. TM led the project. KO performed the immunostaining. RT performed FLX injection, fixation of brains, and data quantification. TI performed the injection of FLX and fixation of brains. KO and TM co-wrote the paper. All authors read and approve the manuscript.

## Supplementary Material

Additional file 1 Figures pertaining to chronic FLX treatment. Click here for file
