# Supplementary material for: Chronic fluoxetine treatment reduces parvalbumin expression and perineuronal nets in gamma-aminobutyric acidergic interneurons of the frontal cortex in adult mice
Source: Mol Brain. 2013 Nov 5;6:43. doi: 10.1186/1756-6606-6-43 (PMC4225860; doi:10.1186/1756-6606-6-43)
Supplement: Additional file 1 — Figures pertaining to chronic FLX treatment. [file 1756-6606-6-43-S1.pdf]

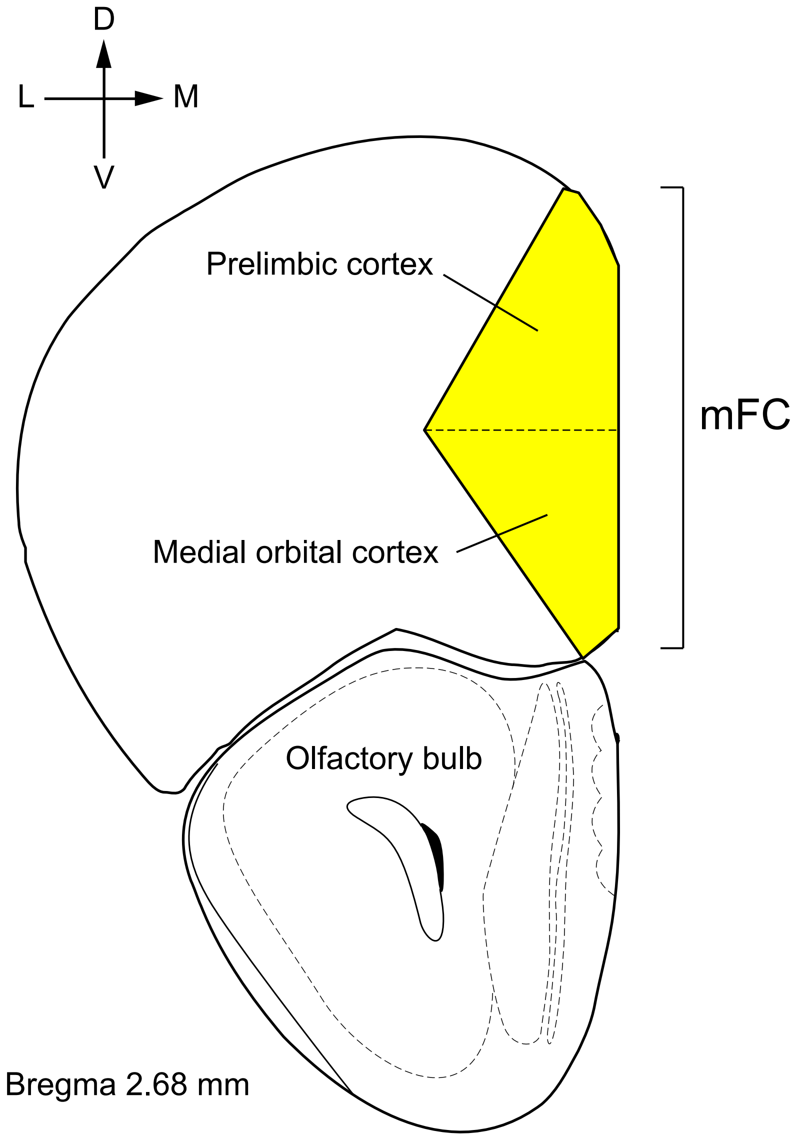

Figure S1. Schematic drawing of coronal sections of the mFC

The mFC (yellow), which we analyzed in this study, contains the prelimbic and medial orbital cortices. mFC, medial frontal cortex.

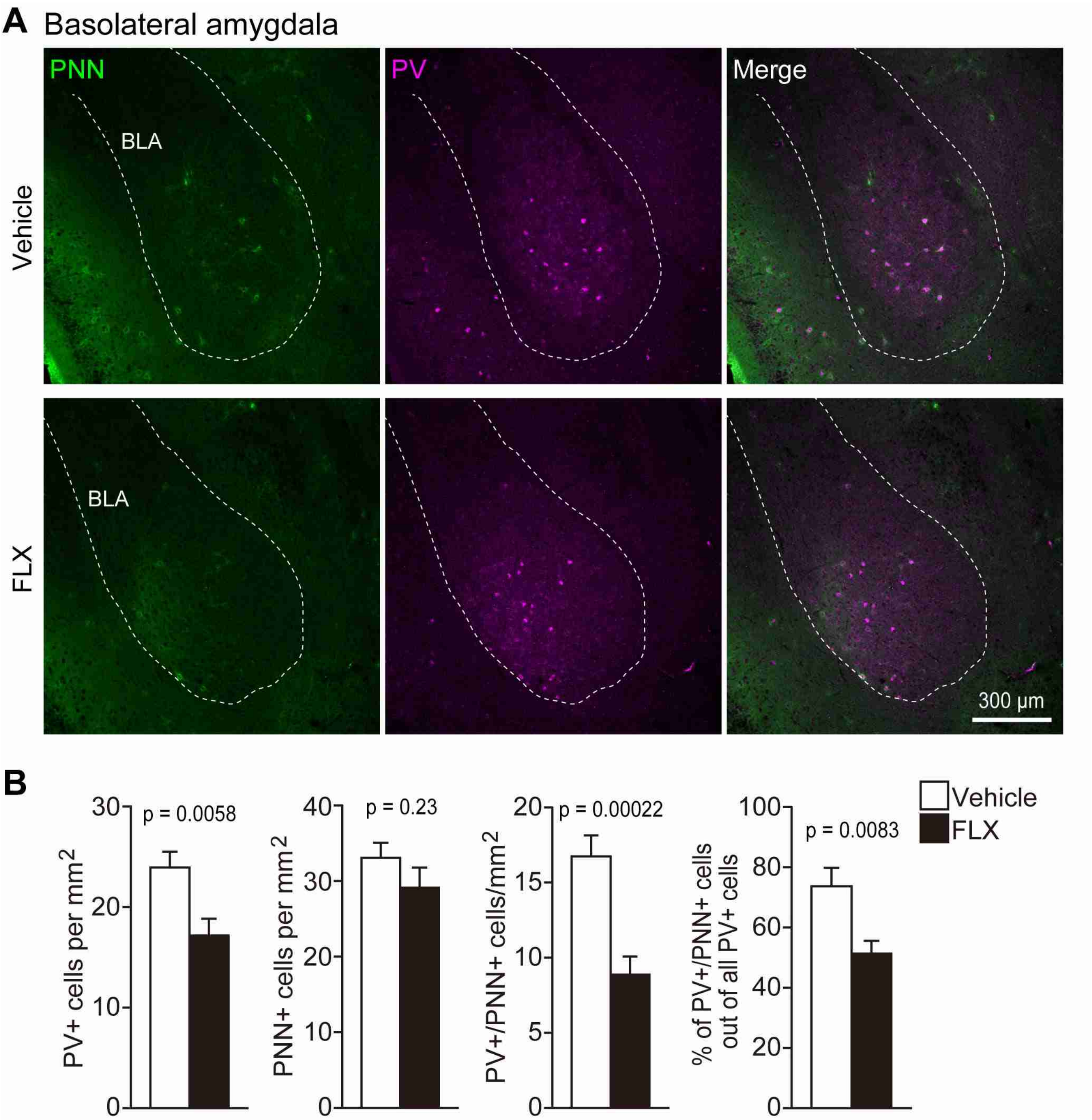

Figure S2. Decrease in the number of parvalbumin+ cells in the basolateral amygdala
(**A**) Representative coronal images of parvalbumin+ (magenta)/PNN+ (green) cells in the basolateral amygdala of mice treated with vehicle (upper row) or FLX (lower row). Mice received FLX for 3 weeks at 15 mg/kg/day. (**B**) Quantification of the number of parvalbumin+, PNN+, parvalbumin+/PNN+ cells, and the proportion of parvalbumin+/PNN+ cells in the total number of parvalbumin+ cells (n = 4 mice each; 11-week-old). BLA, basolateral amygdala; FLX, fluoxetine; PNN, perineuronal net; PV, parvalbumin.

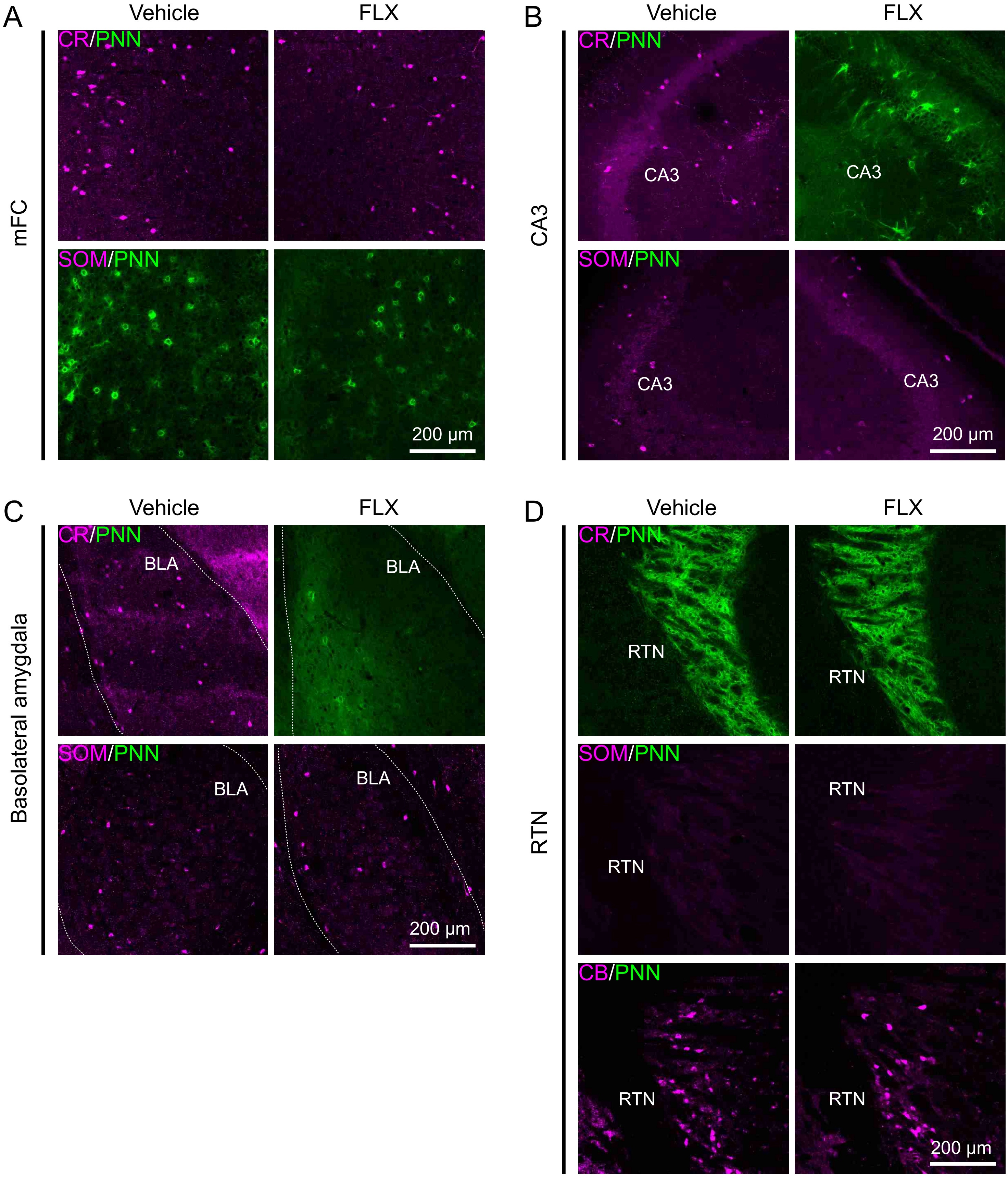
A
Vehicle
FLX
mFC
CR/PNN
SOM/PNN
200 µm
B
Vehicle
FLX
CA3
CR/PNN
CA3
CA3
SOM/PNN
CA3
CA3
200 µm
C
Vehicle
FLX
Basolateral amygdala
CR/PNN
BLA
BLA
SOM/PNN
BLA
BLA
200 µm
D
Vehicle
FLX
RTN
CR/PNN
RTN
RTN
SOM/PNN
RTN
RTN
CB/PNN
RTN
RTN
200 µm

Figure S3

Figure S3. Immunofluorescent staining of interneuron markers and PNN in the mFC, hippocampal CA3, basolateral amygdala, and RTN

(**A-D**) Representative coronal images of double staining for interneuronal markers (magenta) and PNN (green) are shown. Mice received FLX for 3 weeks at 15 mg/kg/day (n = 4 mice each; 11-week-old). Note that calretinin and somatostatin are hardly detected in PNN+ cells in the mFC (**A**), hippocampal CA3 region (**B**), basolateral amygdala (**C**), and RTN (**D**).

BLA, basolateral amygdala; CA, cornu ammonis; CB, calbindin; CR, calretinin; FLX, fluoxetine; PNN, perineuronal net; PV, parvalbumin; RTN, reticular thalamic nucleus; SOM, somatostatin.

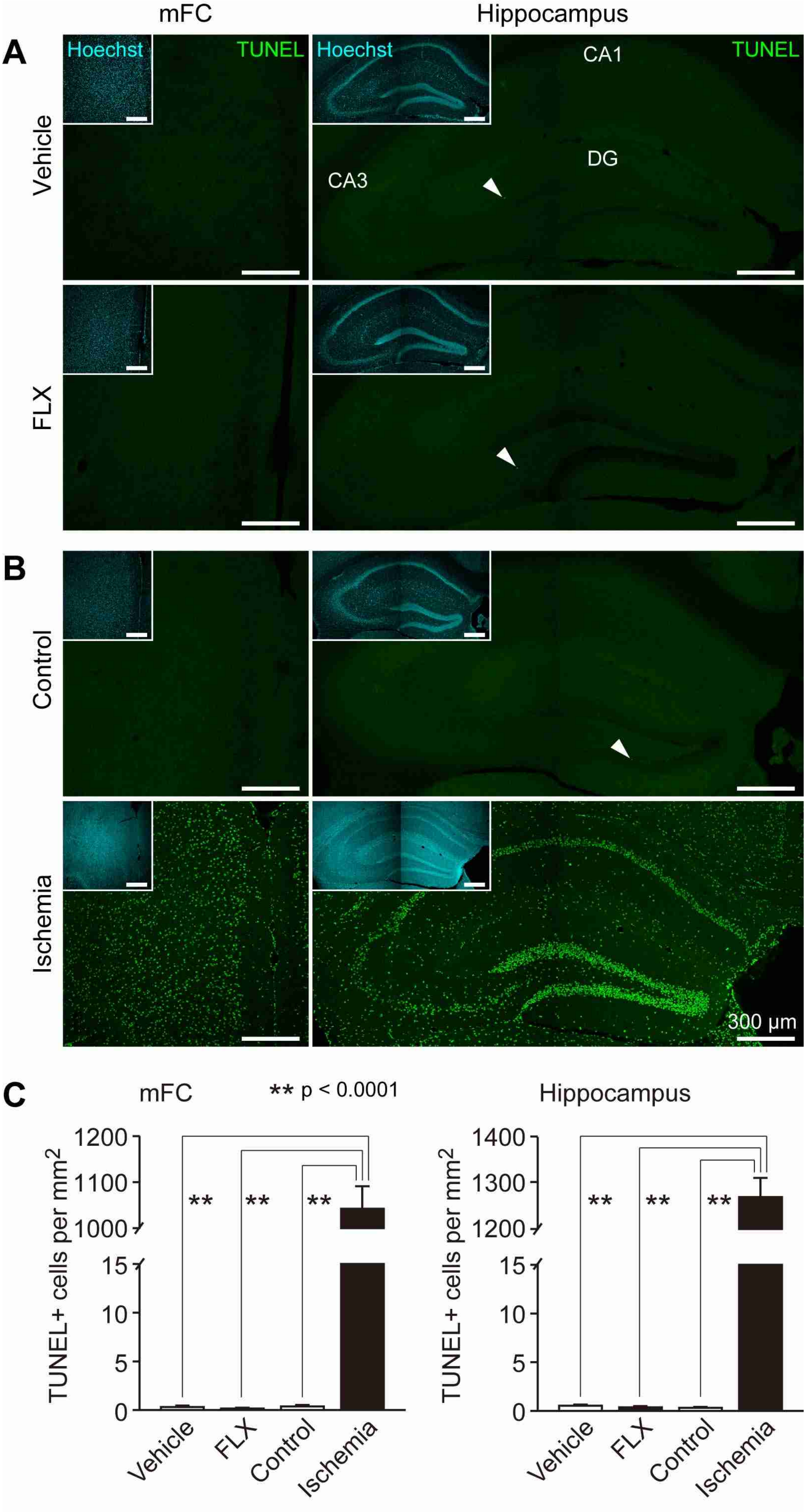
mFC
Hippocampus
A
Hoechst
TUNEL
CA1
DG
CA3
Vehicle
FLX
B
Control
Ischemia
300 µm
C
mFC
** p < 0.0001
Hippocampus
TUNEL+ cells per mm2
1200
1100
1000
1400
1300
15
10
5
0
**
Vehicle
FLX
Control
Ischemia

Figure S4

Figure S4. FLX treatment did not alter the number of apoptotic cells in the mFC and hippocampus

(**A**) Representative coronal images of TUNEL-stained mFC and hippocampal tissues in vehicle or FLX-treated mice. Mice received FLX for 3 weeks at 15 mg/kg/day.

(**B**) Representative images of TUNEL-stained mFC and hippocampal tissues in control or ischemia-treated mice. TUNEL+ cells are indicated by arrowhead (green).

(**C**) Quantification of the number of TUNEL+ cells in (A) and (B) (n = 4 mice each; 11-week-old). CA, cornu ammonis; DG, dentate gyrus; FLX, fluoxetine; mFC, medial frontal cortex; TUNEL, terminal deoxynucleotidyl transferase-mediated dUTP nick-end labeling.

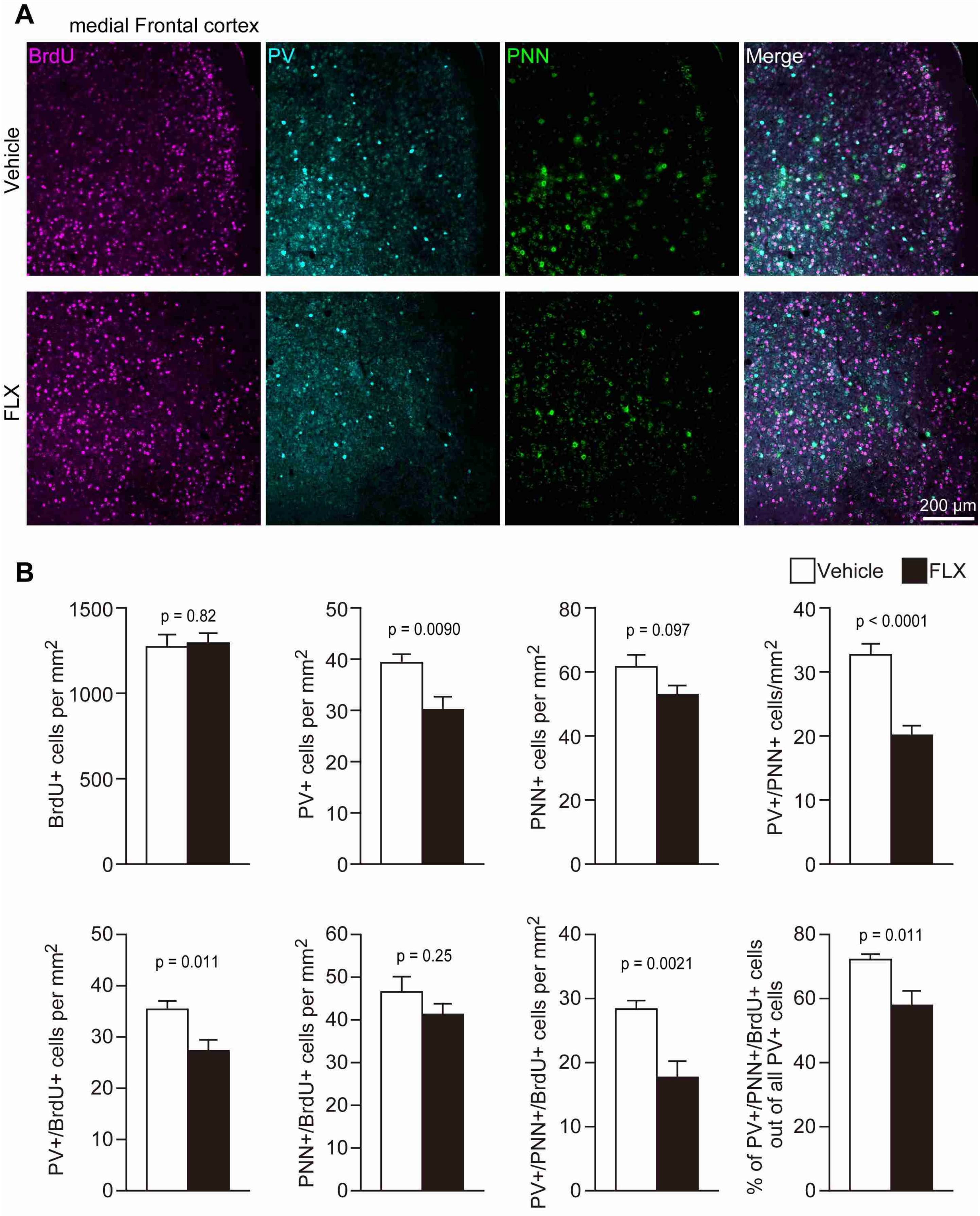
A
medial Frontal cortex
BrdU
PV
PNN
Merge
Vehicle
FLX
200 μm
B
Vehicle
FLX
BrdU+ cells per mm2
p = 0.82
PV+ cells per mm2
p = 0.0090
PNN+ cells per mm2
p = 0.097
PV+/PNN+ cells/mm2
p < 0.0001
PV+/BrdU+ cells per mm2
p = 0.011
PNN+/BrdU+ cells per mm2
p = 0.25
PV+/PNN+/BrdU+ cells per mm2
p = 0.0021
% of PV+/PNN+/BrdU+ cells out of all PV+ cells
p = 0.011

Figure S5

Figure S5. No production of new parvalbumin+ cells with FLX treatments in the mFC

(**A**) Representative coronal images of BrdU- (magenta), parvalbumin- (cyan), and PNN-stained (green) structures in the mFC. Mice received FLX for 3 weeks at 15 mg/kg/day.

(**B**) Quantification of the number of indicated marker-positive cells (n = 4 mice each; 11-week-old). BrdU, 5-bromodeoxyuridine; FLX, fluoxetine; PNN, perineuronal net; PV, parvalbumin.

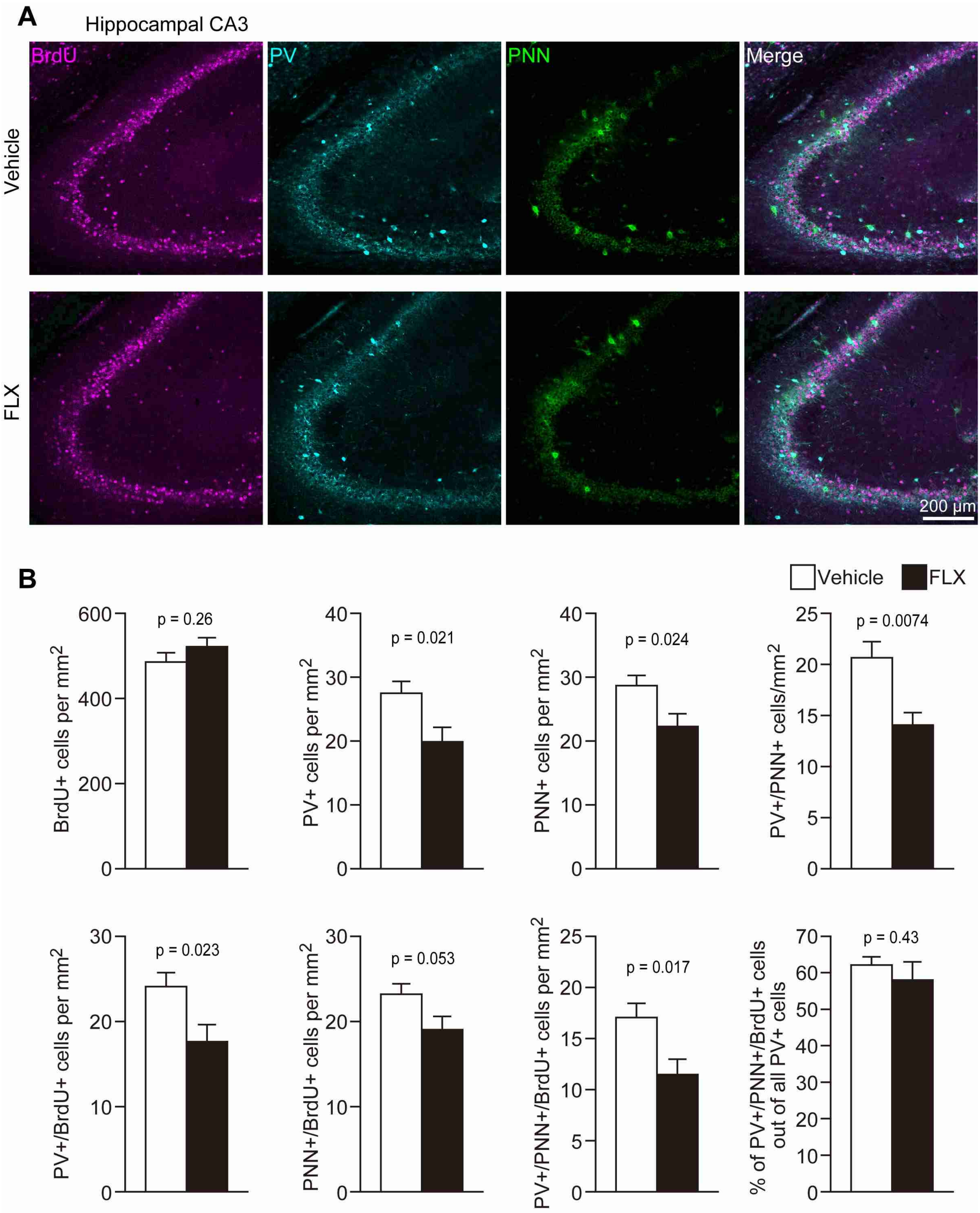
A
Hippocampal CA3
BrdU
PV
PNN
Merge
Vehicle
FLX
200 μm
B
Vehicle
FLX
p = 0.26
BrdU+ cells per mm2
p = 0.021
PV+ cells per mm2
p = 0.024
PNN+ cells per mm2
p = 0.0074
PV+/PNN+ cells/mm2
p = 0.023
PV+/BrdU+ cells per mm2
p = 0.053
PNN+/BrdU+ cells per mm2
p = 0.017
PV+/PNN+/BrdU+ cells per mm2
p = 0.43
% of PV+/PNN+/BrdU+ cells out of all PV+ cells

Figure S6

Figure S6. No production of new parvalbumin+ cells with FLX treatments in the hippocampal CA3 region

(**A**) Representative coronal images of BrdU- (magenta), parvalbumin- (cyan), and PNN-stained (green) structures in the hippocampal CA3 region. Mice received FLX for 3 weeks at 15 mg/kg/day. (**B**) Quantification of the number of indicated marker-positive cells (n = 4 mice each; 11-week-old). BrdU, 5-bromodeoxyuridine; CA, cornu ammonis; FLX, fluoxetine; PNN, perineuronal net; PV, parvalbumin.

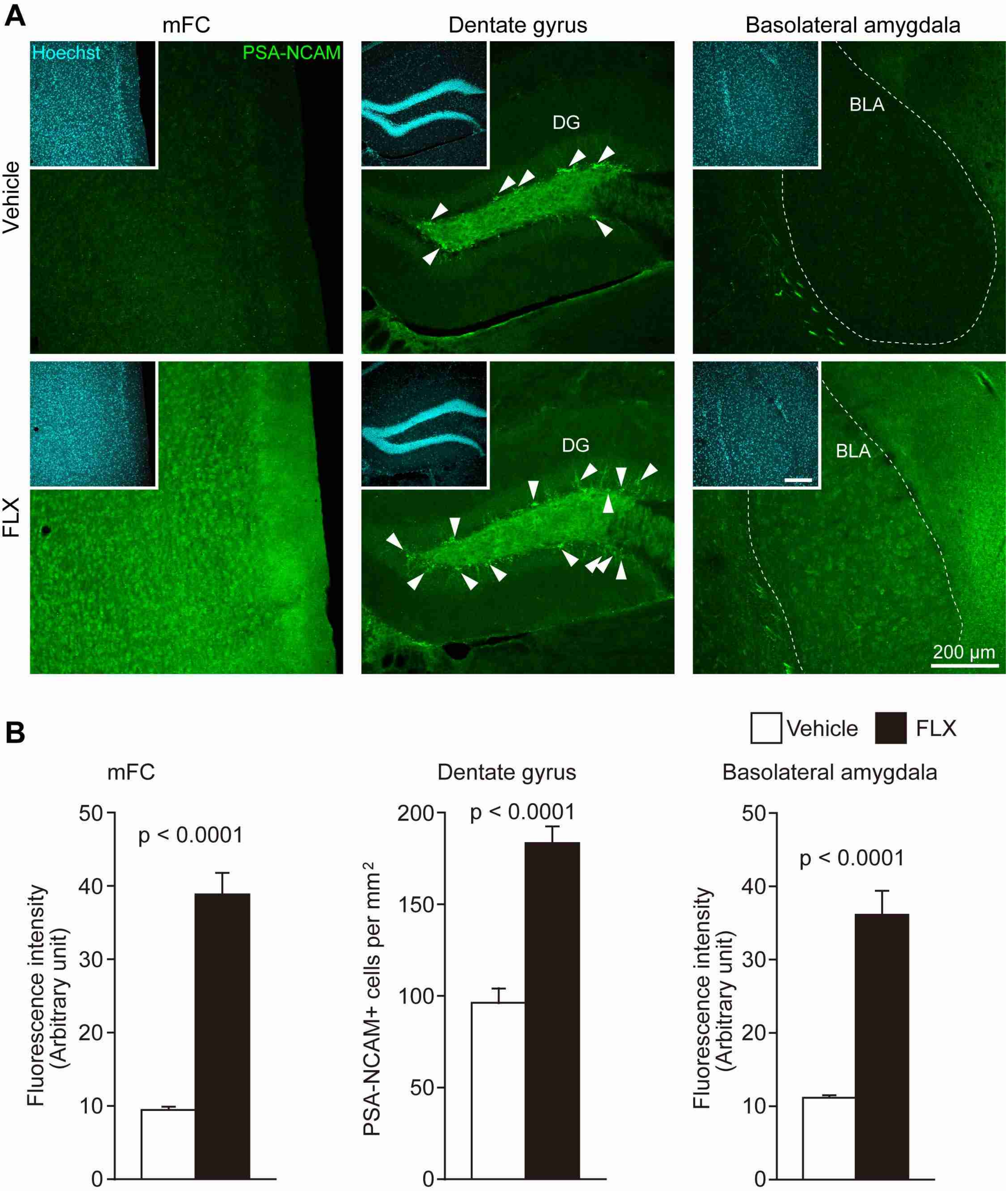
A
mFC
Dentate gyrus
Basolateral amygdala
Hoechst
PSA-NCAM
Vehicle
FLX
DG
BLA
200 µm
B
Vehicle
FLX
mFC
Dentate gyrus
Basolateral amygdala
p < 0.0001
Fluorescence intensity (Arbitrary unit)
PSA-NCAM+ cells per mm2
0
10
20
30
40
50
100
150
200

Figure S7

Figure S7. Increased expression of PSA-NCAM in the mFC, DG, and basolateral amygdala (**A**) Representative coronal images of PSA-NCAM+ structures (green) in the mFC, DG, and basolateral amygdala of mice treated with vehicle (upper row) or FLX (lower row). Mice received FLX for 3 weeks at 15 mg/kg/day. Arrowheads indicate PSA-NCAM+ cells in the DG. Images of the same sections stained with Hoechst are shown in the insets. (**B**) Quantification of the fluorescence intensities of PSA-NCAM signals in the mFC and basolateral amygdala and the number of PSA-NCAM+ cells in the DG of vehicle-treated and FLX-treated mice (n = 4 mice each; 11-week-old). BLA, basolateral amygdala; DG, dentate gyrus; FLX, fluoxetine; mFC, medial frontal cortex; PSA-NCAM, polysialic acid-neural cell adhesion molecule.

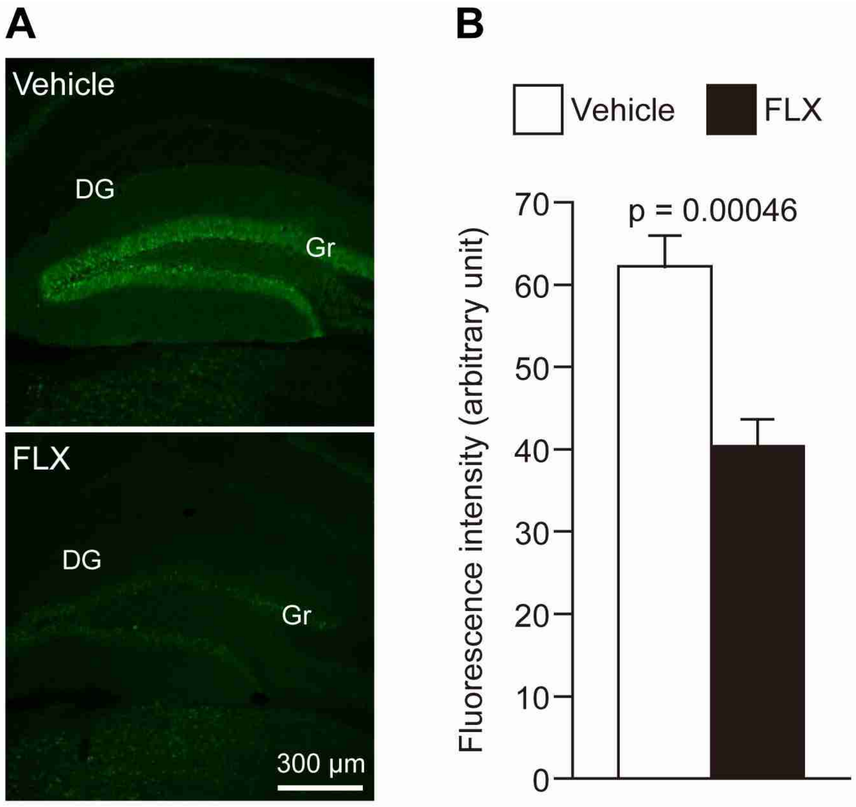

Figure S8. Decreased expression of calbindin in the DG of FLX-treated mice

(**A**) Representative coronal images for the expression of calbindin in the DG of vehicle- (upper) or FLX-treated mice (lower). Mice received FLX for 3 weeks at 15 mg/kg/day. (**B**) Quantification of fluorescence intensity of calbindin in the granule cell layer of the DG in vehicle- or FLX-treated mice (n = 4 mice each; 11-week-old). DG, dentate gyrus; FLX, fluoxetine; Gr, granule cell layer.

Table S1. Density of PNN+ cells containing calretinin or somatostatin in the mFC, CA3, amygdala, and RTN

Region	Marker	Treatment	Marker+ (cells/mm^2)*1	PNN+ (cells/mm^2)	Double+ (cells/mm^2)*2	Double+/total each marker+ (%)*3
mFC	CR	Vehicle	63 ± 2.8	57 ± 2.8	0.38 ± 0.19	59 ± 0.31
		FLX	61 ± 3.0	58 ± 3.3	0.35 ± 0.19	52 ± 0.29
	SOM	Vehicle	47 ± 3.2	58 ± 3.0	0.54 ± 0.26	1.0 ± 0.52
		FLX	43 ± 2.3	54 ± 3.1	0.55 ± 0.29	1.2 ± 0.57
CA3	CR	Vehicle	35 ± 1.8	37 ± 3.7	6.6 ± 0.66	19 ± 2.0
		FLX	32 ± 1.9	38 ± 2.7	5.9 ± 0.72	19 ± 3.3
	SOM	Vehicle	26 ± 2.2	34 ± 1.9	3.1 ± 1.1	11 ± 3.7
		FLX	26 ± 1.1	31 ± 1.6	2.0 ± 0.57	7.3 ± 2.2
Amygdala	CR	Vehicle	72 ± 4.7	38 ± 2.9	0.33 ± 0.33	0.46 ± 0.46
		FLX	65 ± 3.3	38 ± 2.2	0.33 ± 0.33	0.56 ± 0.56
	SOM	Vehicle	26 ± 1.9	31 ± 2.0	0	0
		FLX	26 ± 1.4	28 ± 1.5	0	0
RTN	CR	Vehicle	0.93 ± 0.49	478 ± 17	0	0
		FLX	1.0 ± 0.47	531 ± 26	0	0
	SOM	Vehicle	0.33 ± 0.33	517 ± 17	0	0
		FLX	0.55 ± 0.37	465 ± 28	0	0
	CB	Vehicle	35 ± 5.6	488 ± 23	34 ± 5.5	99 ± 0.97
		FLX	47 ± 7.3	514 ± 21	46 ± 6.9	99 ± 0.71

CA, cornu ammonis; CB, calbindin; CR, calretinin; FLX, fluoxetine; mFC, medial frontal cortex; PNN, perineuronal net; RTN, reticular thalamic nucleus; SOM, somatostatin.

*1: The numbers of marker-positive (+) cells (CR, SOM, or CB) are presented.

*2: The numbers of marker and perineuronal net (PNN) double+ cells are presented.

*3: The percentages of marker and PNN double+ cells out of all marker+ cells are presented.
